# Supplementary figures and images for: Genomic Comparative Analysis of Two Multi-Drug Resistance (MDR) Acinetobacter baumannii Clinical Strains Assigned to International Clonal Lineage II Recovered Pre- and Post-COVID-19 Pandemic
Source: Biology (Basel). 2023 Feb 24;12(3):358. doi: 10.3390/biology12030358 (PMC10045941; doi:10.3390/biology12030358)

*A. baumannii*  
AMA\_NO strain

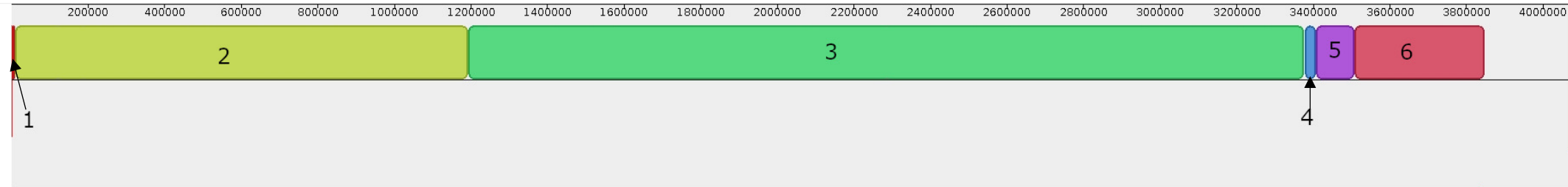

*A. baumannii*  
AMA166 strain

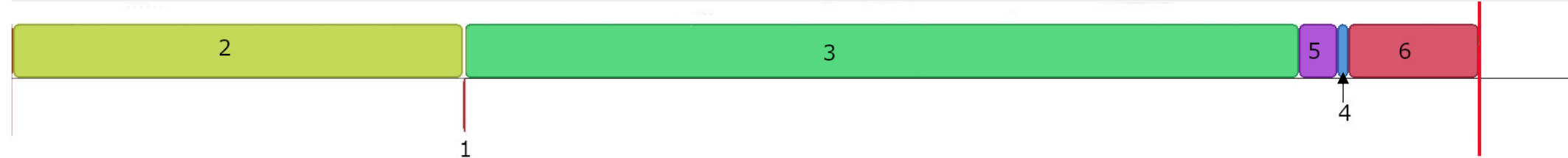

Supplement: Supplementary file 1 [file biology-12-00358-s001.zip › Supplementary Material/Figure_S1.pdf]
